# Evaluation of *Bacillus thuringiensis* Pathogenicity for a Strain of the Tick, *Rhipicephalus microplus*, Resistant to Chemical Pesticides

**DOI:** 10.1673/031.010.14146

**Published:** 2010-10-22

**Authors:** Manuel Fernández-Ruvalcaba, Guadalupe Peña-Chora, Armando Romo-Martínez, Víctor Hernández-Velázquez, Alejandra Bravo de Parra, Diego Pérez De La Rosa

**Affiliations:** ^1^ Centro Nacional de Investigaciones en Parasitología Veterinaria INIFAP. Km. 11.5 Carretera Federal Cuernavaca-Cuautla, Col. Progreso, Jiutepec, Morelos, México, C. P. 62550; ^2^ Centro de Investigaciones Biológicas, Universidad Autónoma del Estado de Morelos, Av. Universidad 1001 Col. Chamilpa, Cuernavaca, Morelos, México, C. P. 62209; ^3^ Centro de Investigaciones en Biotecnología, Universidad Autónoma del Estado de Morelos, Av. Universidad 1001 Col. Chamilpa, Cuernavaca, Morelos, México, C. P. 62209; ^4^ Instituto de Biotecnología, Universidad Nacional Autónoma de México, Av. Universidad 2001 Col. Chamilpa, Cuernavaca, Morelos, México, C. P. 62210

**Keywords:** biological control, ticks, oviposition inhibition, immersion, feeding

## Abstract

The pathogenicity of four native strains of *Bacillus thuringiensis* against *Rhipicephalus* (*Boophilus*) *microplus* (Canestrine) (Acari: Ixodidae) was evaluated. A *R. microplus* strain that is resistant to organophosphates, pyrethroids, and amidines, was used in this study. Adult *R. microplus* females were bioassayed using the immersion test of Drummond against 60 *B. thuringiensis* strains. Four strains, GP123, GP138, GP130, and GP140, were found to be toxic. For the immersion test, the total protein concentration for each bacterial strain was 1.25 mg/ml. Mortality, oviposition, and egg hatch were recorded. All of the bacterial strains had significant effects compared to the controls, but no significant differences were seen between the 4 strains. It is evident that these *B. thuringiensis* strains have a considerable detrimental effect on the *R. microplus* strain that is resistant to pesticides.

## Introduction

In tropical and sub-tropical regions of Mexico, where cattle are raised, the main ectoparasite of economic importance is *Rhipicephalus* (*Boophilus*) *microplus* (Canestrini) (Acari: Ixodidae) (Murrell and Barker 2003), as it causes direct damage by blood feeding and transmitting babesiosis and anaplasmosis (Bram et al. 2002). The control of this tick parasite is based on chemical products. However, *R. microplus* has developed resistance to almost all pesticides used including organophosphates, pyrethroids, and amidines, requiring higher doses or a mixture of several products for their effective control. These practices result in increased production costs and contamination of the environment (Li et al. 2004; Miller et al. 2005).

An alternative is the use of biological control such as the use of predators, parasitoids, and entomopathogens, including fungi, bacteria, viruses, and nematodes. Within the bacterial group, the microorganism most widely used worldwide with the highest success in the control of several insect pests is the bacterium, *Bacillus thuringiensis* Berliner (Bacillales: Bacillaceae). *B. thuringiensis* has been shown to be useful for the control of different insect pests that affect plant crops, forest trees, or that are vectors of human diseases such as dengue and malaria (Crickmore 2005; de Maagd et al. 2003; Schnepf et al. 1998). *B. thuringiensis* represents an important portion of the biopesticides market (Porter et al. 1993), with annual sales around 140 million US dollars and with more than 40% of the sales in the United States (National Academy of Sciences 2003). The use of *B. thuringiensis* is increasing rapidly because it is highly specific, significantly lowering the damage to other organisms compared to use of chemical insecticides, and also because it is biodegradable and is therefore accepted as an environmentally friendly alternative. In addition, *B. thuringiensis* has no adverse effects on humans. *B. thuringiensis* products can be combined with other pest control techniques and it is an essential component in Integrated Pest Management (IPM).

The use of *B. thuringiensis* for cattle tick control has been previously reported (Ostfeld et al. 2006). Hassanain et al. (1997) evaluated the activity of three subspecies of *B. thuringiensis* (*kurstaki, israeliensis,* and *thuringiensis*), spraying spore/crystal mixtures on the soft tick *Argas persicus* and the hard tick *Hyalomma dromedario.* In another report, Samish and Rehacek (1999) mentioned 100% mortality using mixtures of *B. thuringiensis* spores and blood to feed *Ornithodoros erraticus* through an artificial membrane. Zhioua et al. (1999) evaluated a *B. thuringiensis kurstaki* strain against engorged larvae of *Ixodes scapularis,* achieving 96% mortality with a dose of 10^8^ spores/ml.

In this work, the pathogenicity of some native strains of *B. thuringiensis* against a tick *R. microplus* population that is resistant to chemical pesticides was evaluated.

## Materials and Methods

The *B. thuringiensis* strains used in this study belong to the collection of the Vegetal Parasitology Laboratory at the Center of Biological Research at the University of Morelos, Mexico. The GP123, GP138, GP139, GP140 *B. thuringiensis* strains were grown at the University of Morelos's facilities using solid medium Luria-Bertani (LB), until complete sporulation (72 h). Crystal inclusions were observed through an optical phase-contrast microscope. Spores and crystals produced by the *B. thuringiensis* strains were recovered using a bacteriological loop and suspended in 20 ml of sterile water. Finally, the 0.1 mM protease inhibitor (PMSF) was added to avoid protein degradation. Total protein was quantified by the Bradford technique (Bradford 1976).

A *R. microplus* strain resistant to organophosphates, pyrethroids, and amidines was maintained in *Holstein* steers (250 kg weight) at the facilities of INIFAP-CENID-Veterinary Parasitology, at Jiutepec, Morelos, Mexico, where the bioassays were performed. Two steers were artificially infested with 1 g of *R. microplus* larvae. Twenty-one days after infestation, fully engorged female ticks began to drop. Females, weighing 0.2 to 0.4 g, were collected to be used in the bioassay. The adult immersion test developed by Drummond (1969) was used to determine the effect of the *B. thuringiensis* bacterium against *R. microplus* ticks. Engorged adult female ticks were immersed for 60 seconds in a 1.25 mg/ml suspension in water of *B. thuringiensis.* Ticks were then placed individually in 24-well polystyrene plates (Cell Wells, Corning Glass Works, http://www.corning.com/lifesciences). Inhibition of the individual amount of oviposition and egg hatch were recorded during the bioassay. Tick controls were treated with distilled water. Incubation was performed in a humidity chamber (90–95% relative humidity) at 28° C. For each *B. thuringiensis* strain tested, 48 female *R. microplus* ticks were used. Ticks were analyzed under a stereoscope to confirm female tick mortality after 5, 10, 15, and 20 days after inoculation. To measure the effects of bacterial infection on tick fertility and fecundity, an efficiency index was quantified (egg weight/engorged female tick weight) (Drummond 1969). At 10 days after innoculation, egg masses were separated from the female and weighed. The oviposition capacity of control ticks and those surviving the bacterial infection was determined by the efficiency index.

Mortality and egg hatch data were transformed (arcsine) in order to normalize and perform variance analysis (α = 0.05) and mean estimation by using Tukey's test (α = 0.05) and the statistics package SAS 2001. Data obtained from egg weight assessments were not transformed.

## Results

A *R. microplus* strain that is resistant to organophosphates, pyrethroids, and amidines has been used for the assays. As an alternative for the control of this pest, the effectivity of some *B. thuringiensis* strains that were isolated from different insect and arthropod bodies collected from different regions of Mexico were analyzed. Sixty different native *B. thuringiensis* strains were tested, which were only characterized by the presence of a crystal inclusion during bacterial sporulation under phase-contrast optical microscope observations. Among these strains were four native strains that caused mortality in the adult immersion test assay. The mortality induced by strains GP123, GP138, GP139, GP140 on the *R. microplus* adult female was assayed by immersion assay. The immersion assay was first used to determine the toxicity of these four *B. thuringiensis* strains. All of these *B. thuringiensis* strains showed high mortality values statistically different (P< 0.0001) from the controls at all tested times after innoculation. None of the strains were significantly different from one another. The data suggest that GP138 strain had an earlier effect than the other strains (Table 1). The causal agent (the *B. thuringiensis* strains) was recovered from all dead ticks, confirming that *B. thuringiensis* bacteria were responsible for killing the *R. microplus* resistant strain.

The effect of the *R. microplus* strain on oviposition and egg hatch was also analyzed during the immersion trials. Strains GP138, GP139 and GP140 showed similar inhibitory effects without statistically significant differences among them (Tukey's test, α = 0.05) (Table 2), but they were significantly different from the controls.

## Discussion

*B. thuringiensis* are Gram-positive bacteria that are able to produce proteins such as Cry, Cyt, Vip, and S-layer, which have insecticidal properties with different modes of action. These proteins are toxic to insect species belonging to the orders Lepidoptera, Diptera, Coleoptera, Hymenoptera, as well as for acari and nematodes (Bravo et al. 2007; Schnepf et al. 1998; Peña et al. 2006). However, there is a great diversity of arthropod species, such as ticks, for which no specific insecticidal *B. thuringiensis* proteins have been found.

**Table 1.**
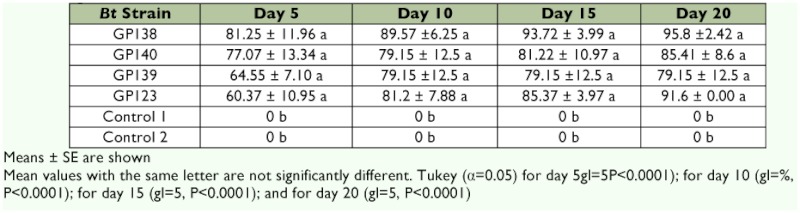
Percentage of *Rhipicephalus microplus* adult female mortality caused by four *Bacillus thurigiensis* strains at different times during immersion trials.

**Table 2.**
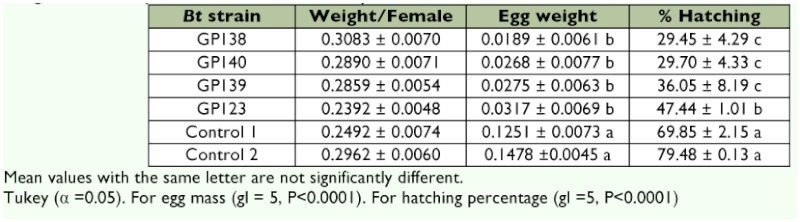
Percentage of female weight, egg weight, and hatching *of Rhipicephalus microplus* females treataed with four *Bacillus thurieiensis* strains by immersion trials after 20 days.

Previous reports about the toxicity of different *B. thuringiensis* strains against ticks are limited. Hassanain et al. (1997) reported that *B. thuringiensis kurstaki* produced 100% mortality against *A. persicus* engorged females after five days at a dose of 1 mg/ml. *B. thuringiensis israelensis* caused 100% mortality at a dose of 2.5 mg/ml, and *B. thuringiensis thuringiensis* at a 5 mg/ml dose induced 93.3% mortality. With *H.* *dromedarii,* none of the *B. thuringiensis* strains produced 100% mortality, even at doses as high as 10 mg/ml. In another report, it was shown that *B. thuringiensis kurstaki* spores (10^6^/ml) were toxic to engorged *I.* *scapularis* larvae. However, an LC50 has been reported with 10^7^ spores (Zhioua et al. 1999). In this work, one dose (1.25 mg/ml) was used for immersion assays to characterize the *B. thuringiensis* strain collection (60 strains). The four selected *B. thuringiensis* strains GP123, GP138, GP139, and GP140 produced 62.5, 81.25, 64.58, and 77.08% mortality, respectively, by the fifth day. These data indicated that the GP138 strain was the most pathogenic. Analysis of the effect of *B. thuringiensis* strains on *R. microplus* with the immersion aassay led us to infer that the *B. thuringiensis* strains can affect *R. microplus* through approaches other than ingestion, probably by means of the spiracles or genital pore as was previously proposed (Zhioua et al. 1999).

It can be concluded that some *B. thuringiensis* strains had a toxic effect on *R. microplus* using the adult immersion assay. The *R. microplus* acaraside-resistant strain could be controlled with pathogenic *B. thuringiensis* strains, however, more studies are necessary to optimize the application of the *B. thuringiensis.* The results indicate that immersion trials are effective to control *R. microplus.*
